# *In situ* degradation of 2-methylnaphthalene by a soil *Penicillium* strain associated with fungal–bacterial interactions

**DOI:** 10.1093/ismejo/wraf260

**Published:** 2025-11-25

**Authors:** Jibing Li, Xixi Cai, Menghui Li, Dayi Zhang, Bei Li, Ling N Jin, Chunling Luo, Gan Zhang

**Affiliations:** State Key Laboratory of Advanced Environmental Technology, Guangzhou Institute of Geochemistry, Chinese Academy of Sciences, Guangzhou, Guangdong 510640, China; Guangdong Provincial Key Laboratory of Environmental Protection and Resources Utilization, Guangdong–Hong Kong–Macao Joint Laboratory for Environmental Pollution and Control, Guangzhou Institute of Geochemistry, Chinese Academy of Sciences, Guangzhou, Guangdong 510640, China; Guangdong Key Laboratory of Ornamental Plant Germplasm Innovation and Utilization, Environmental Horticulture Research Institute, Guangdong Academy of Agricultural Sciences, Guangzhou, Guangdong 510640, China; State Key Laboratory of Advanced Environmental Technology, Guangzhou Institute of Geochemistry, Chinese Academy of Sciences, Guangzhou, Guangdong 510640, China; Guangdong Provincial Key Laboratory of Environmental Protection and Resources Utilization, Guangdong–Hong Kong–Macao Joint Laboratory for Environmental Pollution and Control, Guangzhou Institute of Geochemistry, Chinese Academy of Sciences, Guangzhou, Guangdong 510640, China; University of Chinese Academy of Sciences, Beijing 100039, China; Key Laboratory of Groundwater Resources and Environment, Ministry of Education, Jilin University, Changchun, Jilin 130012, China; College of New Energy and Environment, Jilin University, Changchun, Jilin 130021, China; State Key Laboratory of Applied Optics, Changchun Institute of Optics, Fine Mechanics and Physics, Chinese Academy of Sciences, Changchun, Jilin 130033, China; Department of Civil and Environmental Engineering; Department of Health Technology and Informatics, The Hong Kong Polytechnic University, Hung Hom, Kowloon 999077, Hong Kong, China; State Key Laboratory of Advanced Environmental Technology, Guangzhou Institute of Geochemistry, Chinese Academy of Sciences, Guangzhou, Guangdong 510640, China; Guangdong Provincial Key Laboratory of Environmental Protection and Resources Utilization, Guangdong–Hong Kong–Macao Joint Laboratory for Environmental Pollution and Control, Guangzhou Institute of Geochemistry, Chinese Academy of Sciences, Guangzhou, Guangdong 510640, China; State Key Laboratory of Advanced Environmental Technology, Guangzhou Institute of Geochemistry, Chinese Academy of Sciences, Guangzhou, Guangdong 510640, China; Guangdong Provincial Key Laboratory of Environmental Protection and Resources Utilization, Guangdong–Hong Kong–Macao Joint Laboratory for Environmental Pollution and Control, Guangzhou Institute of Geochemistry, Chinese Academy of Sciences, Guangzhou, Guangdong 510640, China

**Keywords:** fungal bioremediation, emerging contaminant, degradation-related gene, single-cell technology, stable isotope probing

## Abstract

Fungi play critical but underappreciated roles comparing to bacteria in the bioremediation of organic pollutants, particularly emerging contaminants. Numerous fungal species, along with their functional genes and metabolic pathways, remain largely unexplored. Here, we integrate single-cell Raman-activated cell sorting with stable isotope probing to identify and characterize *in situ* active fungi involved in emerging contaminant degradation. This approach enabled the isolation of a *Penicillium* sp. strain LJD-20, previously unreported, which acts as an active degrader of 2-methylnaphthalene, a model emerging pollutant. Genomic analyses revealed that LJD-20 harbors a diverse repertoire of degradation-related genes, including those encoding dioxygenases, methyl hydroxylases, and cytochrome P450 monooxygenases, highlighting its versatile metabolic potential. Single-cell genome sequencing also uncovered a potential close fungal–bacterial co-occurrence, suggesting possible ecological or metabolic interactions. In bioaugmentation trials, strain LJD-20 independently degraded 2-methylnaphthalene and simultaneously promoted the enrichment of other microorganisms involved in its removal. These findings highlight the metabolic versatility and ecological importance of fungi in pollutant degradation and demonstrate the utility of combining single-cell and isotopic approaches to explore microbial function and interaction in complex environments.

## Introduction

In recent years, intensified human activities have led to the accumulation of organic pollutants, particularly emerging contaminants, in the environment. Emerging contaminants refer to synthetic or naturally occurring chemicals, such as pharmaceuticals, personal care products, and industrial compounds, that are not routinely monitored but have the potential to enter ecosystems and cause adverse ecological or human health effects [1]. These pollutants are pervasive, encompassing industrial chemicals, plastics, and personal care products [2]. Along with increasing studies on these pollutants [3, 4], their toxicological consequence attracts increasing attentions owing to their disruptive biomolecular mechanisms, leading to chronic diseases such as reproductive disorders, cardiovascular issues, and cancer [5–7]. Additionally, the efficient removal of these pollutants from the environment has become a focal point of research.

Bioremediation relies on the enzymatic actions of microorganisms to biologically transform or degrade organic pollutants in the environment [8–11]. Though bacteria traditionally lie in the focus of bioremediation studies due to their rapid growth and versatile metabolic pathways, fungi-mediated bioremediation is increasingly recognized for its distinct advantages [7, 12]. Fungi possess extensive mycelial networks, enabling to cover large areas and interact with diverse environmental substrates. They produce enzymes with broad substrate specificity and exhibit resilience to various environmental stressors, which has led to the evolutionary development of stress-adaptive proteins. These proteins, often synthesized or post-translationally modified under stress, include crucial enzymes such as oxidoreductases, peroxidases, cytochrome P450s, and laccases (*Lac*), which are involved in the degradation of organic pollutants [13, 14]. However, only a small proportion of fungi have been characterized in detail, despite that the natural world harboring over 1.5 million fungal species [7]. Each fungal species is estimated to encode averagely 10 345 protein sequences, many of which remain unexplored [7]. This vast diversity suggests exceptional capabilities of fungi for *in situ* bioremediation yet to be identified. Although numerous studies have demonstrated the potential of fungi in bioremediation [15, 16], their roles and applications remain less explored and mechanistically understood compared to bacteria. Fungi can offer distinct advantages in specific environmental contexts, due to traits such as hyphal growth for exploration and resource sharing, robust cell walls for desiccation resistance, and enzymatic systems for degrading recalcitrant polymers like lignin [13, 17]. In addition, interactions with bacteria can further contribute to their metabolic versatility and ecological success [18, 19]. Although fungal–bacterial associations have been described in various biological systems, for instance, in the rhizosphere for plant growth promotion, in lichens enabling survival under extreme conditions, and in marine biofilms facilitating nutrient cycling, their roles in pollutant degradation remain largely unexplored [20–22].

Current knowledge of fungal functional genes and protein sequences primarily stems from laboratory-based pure culture experiments [12, 23, 24]. These techniques can isolate fungi with specific degradation capabilities and elucidate their functional genes. However, the majority of environmental microorganisms are yet to be-cultivated, presenting challenges in identifying the active and important microbes [25–27]. It is particularly crucial for uncovering previously uncharacterized microorganisms capable of *in situ* pollutant degradation. Moreover, although traditional culture techniques are invaluable for studying microbial physiology and interactions under controlled conditions, they may not fully capture the ecological complexity of microbial interactions and degradation activities *in situ* [28–30]. Stable isotope probing (SIP) [31–33] combined with single-cell Raman-activated cell sorting (RACS) provides a powerful platform to identify and isolate pollutant-degrading microorganisms at single-cell resolution [34, 35]. By labeling target pollutants with stable isotopes, this approach enables the identification of active degraders [36, 37]. Raman spectroscopy detects biomolecular signatures in individual cells via light scattering [38] and, when coupled with SIP, can pinpoint isotopic incorporation through spectral peak shifts, thereby linking microbial identity, activity, and functional potential at the single-cell level [39, 40]. Although RACS–SIP has been successfully applied to bacteria in complex environments, such as toluene-degrading or carbon-fixing species [28, 41], its application to fungi and fungal–bacterial associations remains unexplored. To date, no studies have utilized RACS–SIP to identify pollutant-degrading fungi, characterize their associated microbial partners, or analyze their functional genes and metabolic pathways.

In this study, we selected 2-methylnaphthalene, a representative alkylated polycyclic aromatic hydrocarbon (PAH), as a model emerging contaminant due to its environmental persistence and toxicity [42, 43]. Its structure and chemical properties make it representative of a broader class of alkylated PAHs, which are increasingly recognized for their environmental and health risks [44, 45]. This study aimed to identify the active fungal degraders of 2-methylnaphthalene and elucidate their intrinsic metabolic mechanisms. To achieve this, we employed an integrated approach that combined SIP with RACS, followed by genomic and metabolic analyses. This strategy enabled the direct linkage between the metabolic activity of individual fungal cells and their functional identities and genetic features, thereby clarifying their specific contributions to pollutant degradation under environmentally relevant conditions.

## Materials and methods

### Sample collection

Soil samples contaminated with petroleum hydrocarbons were collected from the Shengli Oilfield, China (latitude 37°′N, longitude 118°′E). Upon arrival at the laboratory, part of the soil samples was designated for initial deoxyribonucleic acid (DNA) extraction and chemical analysis, whereas the remaining samples were stored at 4°C for subsequent RACS–SIP experiments. The physicochemical properties of the soil and the concentration of 2-methylnaphthalene are listed in Table S1.

### Microcosm setup

Microcosm experiments were conducted using 150-ml serum bottles. Each bottle was filled with 50 ml of minimal medium (MM; composed of 2.5 g/L K₂HPO₄, 2.0 g/L KH₂PO₄, 3.0 g/L NH₄NO_3_, 0.2 g/L MgSO₄·7H₂O, and 0.1 g/L FeSO_4_·7H_2_O; pH 6.5) and 10 g of soil, supplemented with either unlabeled 2-methylnaphthalene (99%; Hot Ear Technology Co., Ltd., Shanghai, China) or ^13^C-labeled 2-methylnaphthalene (^13^C_11_–2-methylnaphthalene, 99%; Cambridge Isotope Laboratories, Inc., Tewksbury, MA, USA) at an initial concentration of 5 mg/kg. To inhibit bacterial activity and investigate the contributions of fungi and their potential bacterial associations, streptomycin (10 mg/L) was added to the medium. The bottles were incubated under dark conditions at 28°C with shaking at 180 rpm. Samples were collected on days 7 and 14 for chemical analysis. To minimize potential cross-feeding, i.e., the utilization of methylnaphthalene-derived metabolites by non-primary degraders, only day 7 samples were used for SIP-RACS and DNA extraction, as over 95% of 2-methylnaphthalene was degraded by day 14 during bioaugmentation, compared to 59.8% on day 7 (Fig. S1). Accordingly, two experimental treatments were established to characterize active methylnaphthalene-degrading fungi within the complex microbial community: ^12^C_NS (unlabeled 2-methylnaphthalene in native soil treated with antibiotics that inhibit bacteria) and ^13^C_NS (^13^C-labeled 2-methylnaphthalene in native soil treated with antibiotics that inhibit bacteria). In this study, antibiotics exhibited a strong inhibitory effect on bacteria, reducing their abundance by more than 65% at the end of incubation (Fig. S2). Each treatment was performed in six replicates. Additionally, unlabeled 2-methylnaphthalene was added to sterilized soil as a sterile control.

### Acquisition of Raman spectroscopy data from single fungal hyphae

Single fungal hyphae from ^13^C-NS microcosms were analyzed using RACS, with ^12^C controls for comparison. To remove impurities that may interfere with Raman spectroscopy, samples were centrifuged at 2000 g for 5 min. The supernatant was collected, and fungal cells were pelleted by centrifugation at 5000 g for 5 min. The pellets were then washed twice with deionized water to remove potential contaminants, resuspended in deionized water, and deposited (1.5 μl) onto specialized single-cell sorting chips from Hooke Instruments (China). These chips consist of a glass slide coated with a thin aluminum layer, which enables the precise dispensing and positioning of individual cells for subsequent spectral acquisition. Raman spectra were collected using a 532-nm laser over 500–2000 cm^−1^, with 5 mW power and 5 s integration time. Spectral preprocessing included baseline correction and vector normalization. When fungal cells assimilated ^13^C-labeled substrates for biosynthesis, the incorporation of heavier isotopes altered the vibrational energies of chemical bonds, resulting in a redshift in spectral peaks [39, 46]. For instance, the Raman band associated with the protein backbone (Amide III), which typically appears at ~1105 cm^−1^ in unlabeled cells [46], has been reported to shift by ~−23 cm^−1^ upon ^13^C incorporation. These characteristic redshifts enable direct correlation between Raman spectra and ^13^C assimilation, allowing identification of active methylnaphthalene-degrading fungal cells at the single-cell level.

### Identification, sorting, and genomic analysis of ^13^C-Labeled fungal hyphae using Raman-activated cell sorting

To isolate single fungal cells actively involved in 2-methylnaphthalene degradation, we employed RACS. This was performed using the PRECI SCS platform (Hooke Instruments, China), which leverages Laser-Induced Forward Transfer (LIFT) technology [47, 48]. The LIFT process is non-contact and enables direct visualization for precise isolation: a laser pulse irradiates the film on the sorting chip, generating a thrust force that propels a single predetermined cell adhered to the film into a collector [48, 49]. This methodology allows for the selective sorting of individual cells based on their spectral phenotype, following previously established protocols [34, 50]. Cells displaying ^13^C-induced Raman spectral shifts were targeted and individually collected into chips containing cell lysis buffer (Qiagen, Germany). The sorting process, managed by PRECI SCS software, successfully isolated 10 fungal hyphae. Genomic DNA was extracted and amplified from single cells using phi29 DNA polymerase (Qiagen, Germany) via multiple displacement amplification (MDA). The successful DNA amplification was verified by polymerase chain reaction (PCR) targeting the ITS region with primers ITS3F and ITS4R (Table S2). The reactions were set up in a total volume of 25 μl, containing 12.5 μl of 2 × Taq PCR Premix, 1 μl of each primer (10 μM), 0.5 μl of template DNA (20 ng/μl), and nuclease-free water to volume. The amplification was carried out with the following profile: an initial denaturation at 95°C for 5 min; 35 cycles of 95°C for 45 s, 56°C for 45 s, and 72°C for 60 s; culminating in a final extension at 72°C for 7 min to ensure complete product elongation. The successful PCR amplicons were then sequenced on a HiSeq X Ten System (Illumina, USA) employing a paired-end 150 bp (PE150) strategy. To explore the occurrence of fungal-associated bacteria, we amplified and validated bacterial DNA using the 515F/806R primers. To rule out potential procedural contamination, negative controls were included. During single-cell sorting, blank areas (without cells) were selected and subjected to the entire downstream DNA amplification and sequencing protocol alongside the experimental samples. The PCR amplification mixture and thermocycling conditions were identical to those described for the ITS gene analysis. Approximately 4 Gb of sequencing data were generated. Quality control involved filtering short reads (<80 nt) with an average quality score < 25. The clean reads were assembled into high-quality contigs using MEGAHIT [51] and annotated using Prokka v1.12.17 [52]. Metabolic genes associated with 2-methylnaphthalene degradation were identified by aligning sequences to the UniRef90 database using HUMAnN2 [53]. To confirm the presence of fungal-associated bacteria and determine their identity, bacterial genomes were reconstructed through metagenomic binning using the MetaWRAP pipeline [51]. The genomic analysis focused on detecting functional genes related to 2-methylnaphthalene metabolism.

### Deoxyribonucleic acid ultracentrifugation and amplicon sequencing and analysis

To validate the single-cell Raman sorting results, total DNA from ^12^C-NS and ^13^C-NS microcosms was extracted using the Fast DNA Spin Kit for nucleic acid–SIP analysis. Approximately 5 μg of DNA was mixed with TE buffer and CsCl to achieve a buoyant density of ~1.77 g/ml, then subjected to ultracentrifugation at 47500 rpm for 48 hours at 20°C using a Beckman Coulter L-100XP ultracentrifuge, resulting in the separation of “light” (1.7042–1.7089 g/ml) and “heavy” (1.7342–1.7411 g/ml) DNA fractions [54]. Detailed procedures are provided in the Supplementary Materials.

Amplicon sequencing of fungal ITS and bacterial 16S ribosomal ribonucleic acid (rRNA) gene V4 regions was performed on both DNA fractions and RACS-sorted cells using ITS3F/ITS4R and 515F/806R primers, respectively (Table S2). PCR followed established protocols [23], and sequencing was conducted on a HiSeq PE250 platform (Illumina) [50, 55]. Data were processed using QIIME2 (v2019.10.0) and DADA2 [56], and taxonomic assignments were made using the UNITE database for fungal ITS sequences. To determine the fungal taxa responsible for 2-methylnaphthalene degradation and to validate the RACS results, a relative enrichment factor (REF) was calculated as follows [23]:


$$\textrm{REF} =\left(\frac{13C\_ heavt}{13C\_ light}\right)/\left(\frac{12C\_ heavy}{12C\_ light}\right), $$

Where 13C_heavy and 13C_light represent the relative abundances of amplicon sequence variants (ASVs) in the heavy and light DNA fractions from the ^13^C-NS treatment, respectively, and 12C_heavy and 12C_light represent the corresponding values from the ^12^C-NS treatment. Microbial ASVs with relative abundances exceeding 0.5% and REF values greater than 1.5 were identified as active fungal microbes involved in *in situ* 2-methylnaphthalene degradation. These ASVs were further analyzed to identify active fungal taxa involved in 2-methylnaphthalene degradation.

### Cultivation of active methylnaphthalene-degrading fungi, morphological characterization, and growth optimization

To culture active methylnaphthalene-degrading fungi sorted by RACS, potato dextrose broth (PDB; Aoboxing Bio-tech, Beijing, China) [57] was used as the growth medium. During sorting, single fungal hyphae were transferred from the sorting chip to sterile cell receivers containing 4 μl of PDB using the RACS single-cell sorting module. A total of 10 fungal hyphae were successfully sorted and immediately inoculated into the growth medium. After 14 days of incubation at room temperature, the cultures were streaked onto potato dextrose agar (PDA; containing 1.5% agar) plates and incubated for an additional 7 days under the same conditions, followed by a final incubation to obtain pure fungal isolates. Genomic DNA was extracted from the isolates, and their identities were determined by amplifying and sequencing the ITS gene region using ITS3F/ITS4R primers. The amplification and bioinformatics analysis followed the identical protocol as outlined in section “Identification, sorting, and genomic analysis of ^13^C-labeled fungal hyphae using RACS”. We successfully isolated and cultured a fungal strain, designated as LJD-20, which had not been previously reported. The strain has been deposited in the Japan Collection of Microorganisms (JCM37573) and the Guangdong Microbial Culture Collection Center (GDMCC 62387). To assess the presence of associated bacteria, we further amplified bacterial specific 16S rRNA genes using the 515F/806R primer set, and no bacterial DNA was detected in the genome sample of the isolated fungal strain. Morphological features of the isolated fungal strain were examined via electron microscopy. Growth conditions, including temperature (18–38°C) and pH (4–9), were optimized. To confirm the methylnaphthalene degrading capability of LJD-20, degradation experiments were conducted under optimal growth conditions. Experiments were performed in 150-ml amber glass bottles containing 50 ml of MM supplemented with 2-methylnaphthalene at a final concentration of 50 mg/L. The fungal inoculum conditions were consistent with those optimized in the growth experiments. Sterile controls (CK) without fungal inoculation were included. All treatments were conducted in six replicates and incubated at 30°C in the dark with shaking at 180 rpm. Samples were collected on days 0, 3, and 7 for 2-methylnaphthalene extraction and analysis. Details are provided in Supplementary Information.

### Bioremediation potential and bioaugmentation mechanism of active fungi in contaminated soil

Following the SIP-based microcosm protocol, experiments to evaluate the bioremediation potential of fungi in contaminated soil were conducted in 150-ml serum bottles. Three treatments were established: (i) soil supplemented with unlabeled 2-methylnaphthalene and sterilized using a gamma-ray technique, which used as the control (ST), (ii) soil inoculated with LJD-20 and supplemented with unlabeled 2-methylnaphthalene (^12^C_BA), and (iii) soil inoculated with LJD-20 and supplemented with ^13^C-labeled 2-methylnaphthalene (^13^C_BA). The pollutant concentration was consistent with the previous SIP experiment, whereas the fungal concentration was adjusted to ~2 × 10^8^ colony-forming units (CFU)/ml using the dilution plate counting method. Each treatment was performed in six replicates. Chemical analyses were conducted on samples collected at days 7 and 14, as described in the “Chemical Analysis” section. To further investigate the bioaugmentation effects of the introduced fungal strain, additional ultracentrifugation, fractionation, sequencing, and other experiments were performed, as outlined in previous sections. This allowed for a comprehensive understanding of the microbial interactions and functional contributions of LJD-20 in the bioremediation process.

### Chemical and statistical analyses

To evaluate 2-methylnaphthalene degradation and explore microbial metabolic mechanisms, we conducted comprehensive chemical and statistical analyses. For 2-methylnaphthalene quantification in soil and MM medium, samples were extracted with dichloromethane/acetone (1:1, v/v), concentrated under a gentle nitrogen stream, and purified through silica/alumina column chromatography. The purified extracts were then analyzed using GC–MS (Agilent 7890) in NCI–SIM mode, with deuterated PAHs and 2-fluorobiphenyl as internal standards [58]. Metabolites in the MM treatment were identified by LC–MS/MS (AB TripleTOF 6600) after methanol extraction and filtration, using a Waters HSS T3 column and formic acid-modified water/acetonitrile as the mobile phase. Enzyme assays for *Lac*, lignin peroxidase (*LiP*), and manganese peroxidase (*MnP*) were performed using specific commercial kits (Beijing Solarbio Science & Technology Co., Ltd., Beijing, China) according to the manufacturer's instructions. The assays are based on established spectrophotometric methods. Briefly, *Lac* activity was determined by monitoring the oxidation of 2,2-azinobis- (3-ethylbenzthiazoline- 6-sulphonate) (ABTS) at 420 nm. *LiP* activity was measured by the oxidation of veratryl alcohol at 310 nm, and *MnP* activity was quantified by the formation of a Mn^3+^-complex at 465 nm.

Statistical analysis was performed using ANOVA and t-tests (R 4.3.2 and Origin 8.0.25), with *P* < .05 considered significant. All assays were done in triplicate. Metabolomics data processing involved ProteoWizard, XCMS, and SVR normalization, with compound annotation based on in-house/public databases and metDNA. Only features with CV < 0.5 and comprehensive scores >0.5 were retained. The microbial sequencing data and Raman spectra are available in the NCBI and BioStudies databases under accession numbers SUB15473580 and S-MBRS15, respectively. Additional methodological details are provided in Supplementary Materials.

## Results

### Methylnaphthalene degradation efficiency and microbial community dynamics

To assess whether fungi are involved in the 2-methylnaphthalene biodegradation, we established microcosm experiments using native soil with antibiotic treatment to suppress bacterial activity. After 7 days of incubation, the concentration of 2-methylnaphthalene in the native soil treated with antibiotics (NS treatment) decreased from the initial 5 mg/kg to 2.01 mg/kg, resulting in a degradation efficiency of 59.8% (Fig. S1), which was significantly higher than that observed in the sterilized control (4.7 mg/kg). Meanwhile, the added antibiotics effectively suppressed bacteria, reducing their abundance by more than 65% at the end of incubation (Fig. S2). Accordingly, the observed biodegradation was attributed to fungus-assisted processes. A partial loss of 2-methylnaphthalene in the gamma-sterilized control likely resulted from abiotic processes such as photodegradation, volatilization, or adsorption, as the absence of microbial growth confirmed complete sterilization. Additionally, remarkable differences in fungal community structure were observed along degradation (Fig. S3). In original soil, dominant fungal taxa (>5%) included *Fusarium* (27.8 ± 0.53%), *Chaetomium* (13.1 ± 0.01%), *Aspergillus* (5.5 ± 0.03%), and *Cephalotrichum* (5.2 ± 1.17%). After 7 days of incubation, the relative abundance of *Fusarium*, *Chaetomium*, and *Cephalotrichum* decreased to 7.8 ± 0.13%, 7.4 ± 0.86%, and 2.8 ± 0.55%, respectively. On the other hand, other taxa such as *Gibellulopsis* (7.9 ± 1.85%), *Coprinellus* (8.7 ± 0.06%), and *Penicillium* (8.8 ± 0.59%) became dominant. Their relative abundance in original soil was at low level (0.2–0.9%), indicating a significant shift in fungal community structure during degradation process. Bacterial community dynamics are not addressed in this study, as SIP results confirmed minimal participation of bacteria in pollutant degradation under experimental conditions (Fig. S5). Furthermore, our work specifically addressed the roles of fungi and their associations with bacteria in pollutant degradation.

### Single-cell Raman spectroscopy analysis of active methylnaphthalene-degrading microbial cells with ^13^C shifts

**Figure 1:**
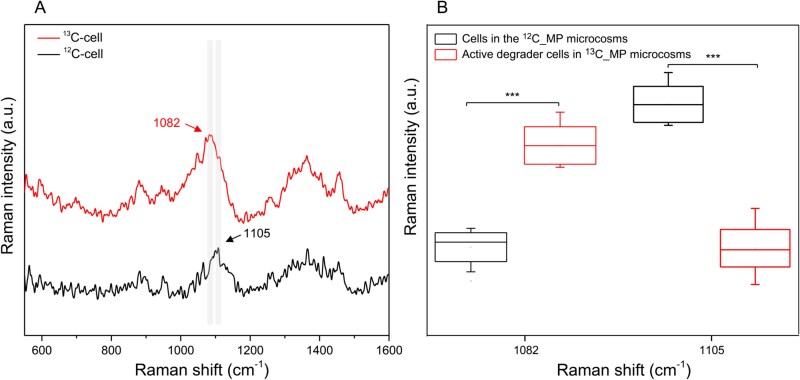
RACS to identify the active methylnaphthalene fungal degraders in soil. (A) Single-cell Raman spectra of cells after *in situ* incubation with ^12^C-NS and ^13^C-NS, tagged as ^12^C-cell and ^13^C-cell, respectively. Each spectrum represents an average of SCRS from 60 detected cells. (B) Intensities of Raman bands at 1105 cm^−1^ and 1082 cm^−1^ based on the active cells detected in ^13^C-NS microcosms and unlabeled cells in ^12^C-NS microcosms. Comparisons denoted by asterisks (***) and a black bar are significantly different (one-way ANOVA, *P* < .001).

To identify the active fungi responsible *in situ* 2-methylnaphthalene degradation in contaminated soil, single-cell Raman spectroscopy (SCRS) was performed on 150 single fungal hyphae from ^13^C-NS microcosm. Among them, 10 fungal hyphae exhibited unique Raman spectral bands with specific ^13^C shifts that were absent in cells from ^12^C-NS microcosm (Fig. 1A). In unlabeled microbial cells, a common Raman band at 1105 cm^−1^, representing Amide III biomarker, shifted to 1082 cm^−1^ in the active methylnaphthalene-degrading cells from ^13^C-NS microcosm. Additionally, Raman intensity analysis revealed distinct differences between the active cells from ^13^C-NS and ^12^C-NS treatment (*P* < .001) (Fig. 1B). These findings demonstrated that the active methylnaphthalene-degraders could assimilate and incorporate ^13^C isotopes into biomolecules, and confirmed the ability of SCRS to accurately identify functional fungal cells with ^13^C-shifted spectral bands, enabling precise detection of the active methylnaphthalene-degrading microbes.

### Identification of active *in situ* methylnaphthalene-degrading microbes and validation by deoxyribonucleic acid–stable isotope probing

**Figure 2:**
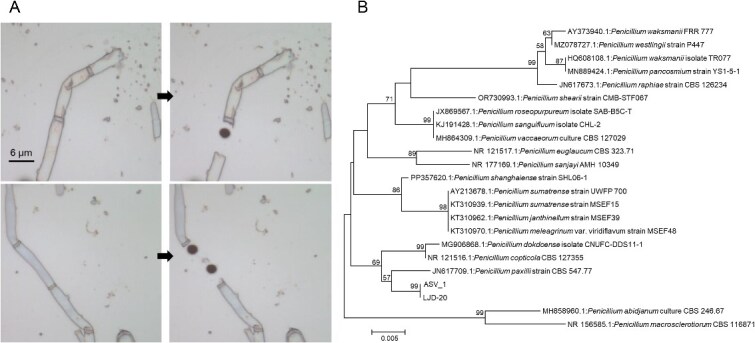
RACS-based isolation and phylogenetic characterization of functional fungal cells. (A) Active fungal hyphae on the sorting chip were identified by SCRS from ^13^C-NS microcosms (left), and cells were ejected off the sorting chip by RACS (right). Images were captured using reflective bright-field imaging. The black dots indicate the locations where the Raman laser interacted with the metal-coated substrate during cell sorting, resulting in partial ablation of the coating and forming visible marks. (B) Phylogenetic tree of RACS-identified ASV based on its ITS gene sequence. Bootstrap values (expressed as percentages of 1200 replications) > 50% are shown at the branch points. Bar: 0.005 substitutions per nucleotide position.

**Figure 3:**
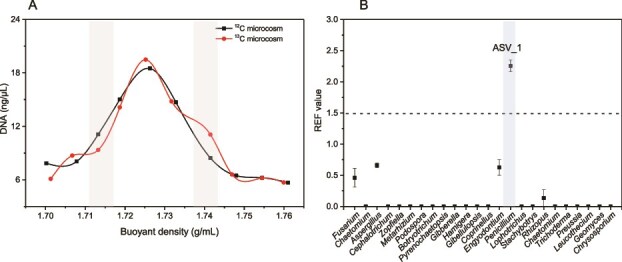
Identification of heavy DNA and tracing of functional microorganisms using nucleic acid–stable isotope probing. (A) Correlations between DNA concentration and buoyant density (BD, g/ml) in DNA extracted from ^12^C_NS and ^13^C_NS microcosms. Heavy-DNA fraction is highlighted. (B) The enrichment factor (REF) of fungal ASVs from NS treatments.

The identified active fungal hyphae were further sorted by RACS (Fig. 2), and the MDA products were subjected to ITS gene and single-cell genome sequencing after single-cell amplification. To ensure sufficient sequencing depth, DNA from all RACS-sorted single cells was pooled for single-cell genome sequencing. ITS sequencing results identified an active fungus involved in 2-methylnaphthalene degradation, classified as *Candidatus Penicillium* sp. Its sequence exhibited the highest similarity (96.7%) to *Penicillium paxilli* strain CBS 547.77 (GenBank accession JN617709.1), which is below the threshold (98.6%) defining microbial species (Fig. 2B). Consequently, we proposed this strain as a previously unreported fungal species and designated it as *Penicillium* sp. LJD-20. To verify the methylnaphthalene-degrading role of *Penicillium* sp. LJD-20 identified by RACS, we performed DNA–SIP analysis. Fungi that incorporated ^13^C-labeled 2-methylnaphthalene into their DNA were enriched in the heavy fraction. ASV_1 showed the highest enrichment, with a REF of 2.3 [57], indicating active involvement in 2-methylnaphthalene degradation (Fig. 3). The ITS sequence of ASV_1 was 100% identical to that of LJD-20, and both clustered phylogenetically with *P. paxilli* (Fig. 2B), confirming that LJD-20 is the active degrader *in situ*. Additionally, we also attempted to explore whether bacteria contributed to 2-methylnaphthalene degradation, and SIP results showed that bacteria did not participate in 2-methylnaphthalene degradation within this experimental system with the supplement of antibiotics (Fig. S4). REF values of all bacterial taxa were <1.5, and our results suggested that 2-methylnaphthalene degradation was primarily driven by fungi rather than bacteria.

### Discovery of a potential fungal–bacterial co-occurrence and its possible ecological implications

Single-cell genomic analysis unexpectedly detected the co-occurrence of bacterial genomic sequences with fungal DNA. Taxonomic analysis identified *Achromobacter* sp. as the dominant bacterium, comprising 92.6% of all bacterial reads. This bacterium was absent in DNA extracts from the procedural negative controls (blank sorts without a cell target), which rules out contamination introduced during the single-cell sorting or amplification processes. Genome binning further reconstructed a 51% complete bacterial genome (Table S4). However, SIP results and antibiotic treatments suggest that the bacterium does not directly contribute to 2-methylnaphthalene degradation, suggesting its functional role may lie outside this specific process.

**Figure 4:**
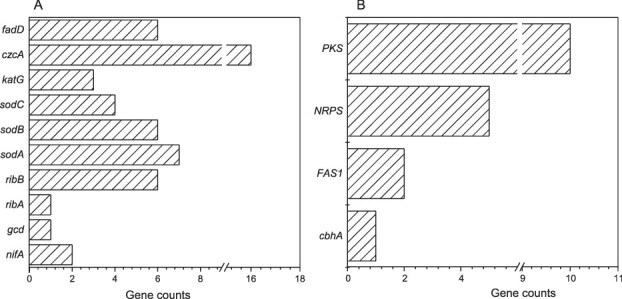
Genomic insights into the metabolic potential of sorted microbial cells. (A) Gene counts related to nitrogen fixation, phosphate solubilization, vitamin biosynthesis, stress resistance, heavy metal detoxification, and fatty acid utilization in bacterial genomes. (B) Gene counts related to carbohydrate and organic acid metabolism, lipid metabolism, and antibiotic biosynthesis in fungal genomes.

Functional gene analysis revealed that the bacterium harbors individual genes absent in the fungus, including those associated with nitrogen fixation (e.g. *nifA*), phosphate solubilization (*gcd*), vitamin biosynthesis (*ribA*, *ribB*), oxidative stress resistance (*sodA*, *sodB*, *sodC*, *katG*), and heavy metal detoxification (*czcA*) (Fig. 4).The *nif* genes encode nitrogenase and its regulatory proteins, with *nifA* activating nitrogen fixation gene clusters. The *gcd* gene encodes glucose dehydrogenase, which catalyzes the production of gluconic acid from glucose; this process can acidify the microenvironment and solubilize inorganic phosphate compounds, thereby enhancing phosphate bioavailability [59, 60]. The *rib* operon supports riboflavin biosynthesis, with *ribA* catalyzing the initial step and *ribB* promoting precursor conversion. Additionally, *SOD* genes (including *sodA*, *sodB*, and *sodC*) and *katG* enhance oxidative stress resistance, whereas *czcA* encodes a cobalt-zinc-cadmium efflux system for heavy metal detoxification.

Genomic analysis revealed that the fungus possesses genes related to cellulose degradation (e.g. *cbh1*) and fatty acid synthesis (*fas1*), whereas the associated bacterium harbors genes potentially involved in fatty acid metabolism (*fadD*). These findings raise the possibility that the fungus may produce metabolites, such as glucose and fatty acids, which could serve as substrates for the bacterium. Moreover, fungal biosynthetic gene clusters (*nrps*, *PKS*) encode antimicrobial compounds, including lipopeptides, macrolides (e.g. erythromycin, avermectin), and bacteriocins (e.g. mycobacillin, weinberg mycin), potentially suppressing microbial competitors (Fig. 4B).

### Cultivation of active methylnaphthalene-degrading fungi and assessment of methylnaphthalene degradation in minimal medium and soil

**Figure 5:**
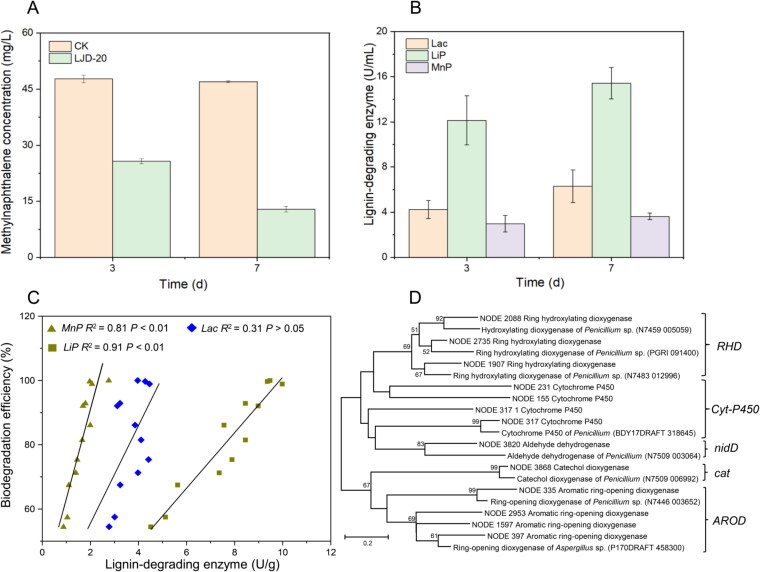
Metabolic potential of isolated microorganisms and analysis of their extracellular enzymes. (A) Biodegradation of 2-methylnaphthalene by strain LJD-20 after 7 days of incubation. Data are means of three replicates. (B) *Lac*, *LiP*, and manganese proxidase (*MnP*) activities of strain LJD-20 in potato dextrose broth after 7 days of incubation. (C) Pearson correlation coefficients between methylnaphthalene degradation and enzyme activities in soil treatments. (D) Phylogenetic tree of *RHD*, aromatic *AROD*, *nidD*, and Cyt-P450 genes detected from RACS-sorted cells.

To cultivate the active fungal degraders sorted by RACS, we used sterile PDB medium and successfully isolated the target strain *Penicillium* sp. LJD-20. After 7 days of cultivation on PDA medium, the colonies appeared flat with a radially arranged pattern, measuring 30–35 mm in diameter (Fig. S5). The texture was fluffy, with soft hairs at the center, neat edges, white mycelia, and abundant light-green conidia. The underside of the colonies was brown. After multiple rounds of cultivation, 16S rRNA gene amplification and sequencing of total fungal DNA revealed no trace of the bacterium. The fungal strain demonstrated satisfactory growth across a temperature range of 18–38°C, with optimal growth at 30°C. It also grew across a pH range of 4–9, with pH 6 being optimal (Fig. S6). Under these optimal growth conditions, 2-methylnaphthalene degradation capability of strain LJD-20 was assessed in both MM medium and native soil. After 7 days of cultivation in MM, the concentration of 2-methylnaphthalene decreased from 50.0 mg/L to 25.8 mg/L in the LJD-20 treatment, whereas it remained at 47.7 mg/L in the control group (CK, Fig. 5A). In contaminated soil, LJD-20 also demonstrated excellent 2-methylnaphthalene degradation performance at ambient temperature. The degradation of 2-methylnaphthalene in treatments bioaugmented with LJD-20 (BA treatments) decreased from the initial 5 mg/kg to 1.2 mg/kg after 7 days of incubation, which was significantly lower than 2.0 mg/kg observed in treatments without fungal supplement (NS treatments; *P* < .05, Fig. S1). Furthermore, after 14 days, the 2-methylnaphthalene concentration in NS treatments decreased to 0.5 mg/kg, whereas LJD-20 inoculation led to complete removal. These results demonstrate the potential of the RACS-sorted fungus LJD-20 in degrading 2-methylnaphthalene in soil.

### Enzyme activities of fungi in soil

To further investigate the mechanisms of *in situ* 2-methylnaphthalene degradation by strain LJD-20, we analyzed its enzyme production and evaluated enzyme activities in different treatments. Stain LJD-20 exhibited the ability to produce three key enzymes: *Lac*, *LiP*, and *MnP*. After 7-day cultivation in MM medium, enzyme activities ranked as *LiP* > *Lac* > *MnP*, with *LiP* activity reaching 15.4 U/ml (Fig. 5B). In soil treatments, the introduction of LJD-20 significantly increased the activities of all these fungal enzymes. After 14-day incubation, *Lac*, *LiP*, and *MnP* activities in LJD-20-inoculated BA treatment reached 4.2, 9.6, and 2.3 U/g, respectively, 15.3–24.5% higher than control group. These results suggest that LJD-20 exhibits a capacity for enzyme production potentially involved in 2-methylnaphthalene degradation (Fig. S7). Subsequently, we analyzed the correlations between soil enzyme activities and 2-methylnaphthalene removal efficiency. The results revealed significant positive correlations between 2-methylnaphthalene degradation and the activities of *LiP* (*R*^2^ = 0.91, *P* < .01) and *MnP* (*R*^2^ = 0.81, *P* < .01) (Fig. 5C). Among them, *LiP* showed the strongest correlation with pollutant biodegradation. These findings suggest that these enzymes may play an important role in the 2-methylnaphthalene degradation process, with *LiP* potentially contributing more prominently.

### Linking genotype and function of active fungi and elucidating their methylnaphthalene metabolic mechanisms in soil

**Figure 6:**
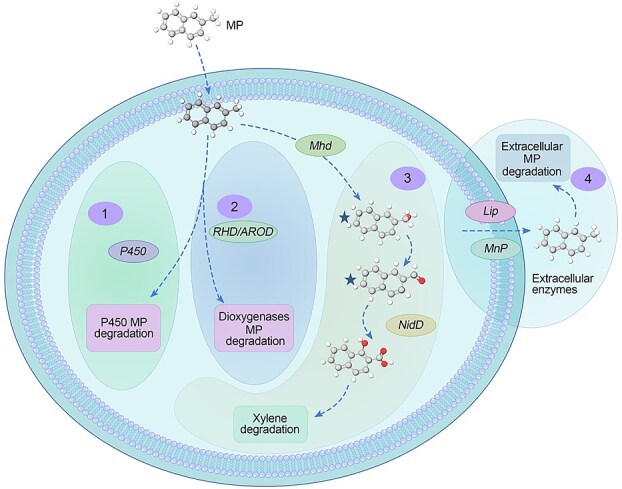
Reconstruction of methylnaphthalene metabolic pathway in sorted cells incorporating ^13^C-NS based on metabolic genes and metabolites, characterized by SIP-RACS. Two starred compounds represent key detected metabolites of 2-naphthalenemethanol and 2-naphthaldehyde. *Mhd* is proposed as a potential methylnaphthalene-degrading gene, and others including *RHD*, *AROD*, *nidD*, and *Cyt-P450* were identified in single-cell genomes.

To elucidate 2-methylnaphthalene biodegradation and bioaugmentation mechanisms of the active fungal degrader identified by RACS, we analyzed their functional genes and potential metabolic pathways through single-cell genome sequencing and annotation using the KEGG database. In addition to detecting genes encoding commonly observed cytochrome P450 enzymes in fungi (Fig. 5D), we identified dioxygenase genes typically associated with bacteria, which are critical for aromatic compound degradation. These included genes encoding ring-hydroxylating dioxygenases (*RHD*), aromatic ring-opening dioxygenases (*AROD*), and aldehyde dehydrogenase (*nidD*), a key enzyme converting 2-naphthaldehyde to 2-naphthoate during methylnaphthalene metabolism (Figs 5D and 6). Functional annotation and sequence homology indicate that the identified genes are likely of fungal origin. The identified dioxygenases were previously uncharacterized and exhibited relatively strong amino acid sequence similarity to previously reported proteins. For instance, *RHD* exhibited sequence similarity of 78.3% and 78.1% to proteins from *Penicillium* sp. (N7446_003652) and *Aspergillus* sp. (P170DRAFT_458300), respectively (Fig. 5D); *AROD* showed sequence similarities of 76.7%, 76.2%, and 73.6% to proteins from *Penicillium* sp. strains N7483_012996, N7459_005059, and PGRI_091400, respectively. The identified P450 enzymes exhibited less than 83.4% amino acid sequence similarity to previously characterized proteins, indicating they are divergent homologs within known P450 families. Similarly, *nidD* observed in this work had a sequence similarity of less than 80% to its closest known counterparts. These findings suggested that the methylnaphthalene-degrading strain LJD-20 harbored functional proteins encoded by genes associated with organic pollutant removal. Based on the genomic and extracellular enzyme data, we hypothesize that strain LJD-20 might possess the genetic potential for multiple metabolic strategies when exposed to 2-methylnaphthalene (Fig. 6). This represents a preliminary insight that requires further validation to confirm the actual degradation pathways and their regulation.

Introduced into soil, LJD-20 did not only degrade 2-methylnaphthalene through intrinsic mechanisms but its presence was also associated with the emergence of other microbes participating in 2-methylnaphthalene removal. The SIP results in BA treatments revealed additional functional fungi involved in 2-methylnaphthalene degradation through REF analysis of the heavy- and light-DNA fractions. In addition to LJD-20 (represented by ASV_1, REF = 2.0), another fungus (*Aspergillus* sp., represented by ASV_12, REF = 3.9) also contributed to 2-methylnaphthalene degradation (Fig. S8). This finding broadens our understanding of pollutant removal mechanisms by revealing a diversity of fungal taxa capable of this process, thereby supporting their consideration in bioaugmentation strategies for environmental remediation. By linking the functions of the active methylnaphthalene-degrading fungi to their genotypes, we constructed a putative comprehensive map of 2-methylnaphthalene metabolic pathway (Fig. 6), thereby enhancing our understanding of their roles in 2-methylnaphthalene degradation.

### Elucidation of potential fungal methylnaphthalene metabolic pathways using liquid chromatography-tandem mass spectrometry

LC–MS/MS analysis of strain LJD-20 in MM during 2-methylnaphthalene biodegradation revealed two major intermediate metabolites: 2-naphthalenemethanol and 2-naphthaldehyde. These metabolites were selected as core candidates based on consistent detection across replicates. By integrating these chemical profiles with genomic evidence, we proposed potential metabolic pathways (Fig. 6). The detection of 2-naphthalenemethanol and 2-naphthaldehyde indicates the potential for a methyl-terminal oxidation process, which may involve methylhydroxylase (*mhd*) activity. Supporting this interpretation, a *nidD*-like gene encoding an aldehyde dehydrogenase was identified in the fungal genome through single-cell sequencing, suggesting a potential enzymatic role in side-chain oxidation during 2-methylnaphthalene degradation.

Taken together, the genomic analysis of *Penicillium* sp. LJD-20 suggests a genetic potential for diverse metabolic functions in the presence of methylnaphthalene. Genes associated with both ring-cleavage and methyl oxidation suggest hypothetical pathways for 2-methylnaphthalene metabolism (Fig. 6). Although the associated genes are not organized within a single operon or gene cluster, the presence of diverse yet functionally relevant genes points to metabolic versatility. Rather than representing fully parallel or redundant pathways, this diversity may reflect ecological plasticity, enabling the fungus to respond to varying substrate concentrations and environmental conditions. Overall, these findings indicate the existence of partially overlapping degradation strategies that contribute to the organism’s adaptive capacity.

## Discussion

Fungi play an important role in the degradation of organic pollutants, particularly recalcitrant and emerging contaminants [7, 61]. However, numerous fungal species and their associated microbial consortia, along with their enzymes and functional genes, remain unexplored. Our study introduced an approach for identifying, sorting, cultivating, and analyzing the degradation pathways and mechanisms of previously uncharacterized fungi, providing a framework for future studies. The research expectation is to inspire further investigation into fungal contributions to pollutant degradation.

This study represents an integration of single-cell RACS with SIP in the field of fungal microbiology. Our approach enables simultaneous identification and functional characterization of fungal degraders and their closely associated bacteria, significantly advancing our understanding of pollutant degradation by fungal-centered consortia. These findings were further validated by nucleic acid-based SIP, supporting their reliability. Our data reveal a dominant role for fungi in contaminant degradation, whereas the associated bacteria appear to play a limited direct role. This observation challenges traditional bacteria-centric models of bioremediation and highlights the ecological importance of fungi in such processes. Although our data do not demonstrate a symbiotic relationship, genome-based analyses suggest the presence of tightly associated bacterial partners that may contribute to fungal fitness through metabolic complementarity, such as nitrogen fixation, phosphate solubilization, and vitamin biosynthesis, whereas fungi may provide carbon sources like sugars and fatty acids. This potential interaction could enhance the resilience of fungal–bacterial consortia under environmental stress. Similar cooperative interactions have been reported in other fungal–bacterial systems [18]. For example, *Bacillus pumilus* contributes fixed nitrogen to *Ustilago maydis*, whereas *Nostoc punctiforme* supplies photosynthates to its fungal host Geosiphon pyriformis in exchange for minerals and CO₂ [62, 63]. Despite these precedents, the biological significance and functional roles of fungal–bacterial systems remain poorly understood, particularly in the context of secondary metabolism and environmental adaptation. In this study, we provide genomic-level evidence indicating a potential fungal–bacterial association that may be relevant to environmental remediation. Although our single-cell Raman and genomic analyses offer higher resolution and specificity than traditional metagenomics, we fully acknowledge that further structural and functional validation, such as through transmission electron microscopy or co-culture experiments, is required to confirm the nature of this association.

Our study identified the fungus *Penicillium* sp. LJD-20, which carries genes related to methyl-terminal oxidation and oxygenase-mediated reactions, potentially involved in 2-methylnaphthalene degradation *in situ*. These capabilities distinguish it from previously reported fungal degraders. Isolating such an *in situ* functional fungus is particularly challenging, especially for uncharacterized strains from complex environments. Traditional fungal bioremediation studies rely on pure culture techniques to isolate highly efficient degraders under controlled laboratory conditions [5, 64]. However, these strains often fail to adapt to real-world remediation scenarios due to environmental stresses and microbial competition [23, 65]. Although SIP inherently requires incubation and thus represents a form of cultivation, its integration with single-cell RACS allows direct detection and isolation of active degraders at the single-cell level. This approach substantially narrows the enrichment window and reduces cultivation bias, improving the capture of environmentally relevant microorganisms. Bioaugmentation results further demonstrated the effectiveness of LJD-20 in enhancing pollutant removal by indigenous microbes in contaminated soil. These findings provide insights into the isolation of fungal degraders and their potential application in bioremediation, helping in method improvement for traditional approaches screening microbes responsible for emerging contaminant biodegradation.

Another contribution of this study is the direct linkage of fungal functions to their genotypes and potential metabolic pathways at the single-cell level. Previous research on functional fungi has primarily focused on abundance, extracellular enzymes, and cytochrome P450 activities [7, 23]. For studies using community-level functional approaches, such as SIP, to investigate the composition of the active fungal communities, they fail to link fungal identities with their functional genes or metabolic pathways [13, 57]. This limitation has hindered the in-depth understanding of pollutant removal mechanisms. In this study, we employed single-cell sorting and genome sequencing technologies to bridge this gap, linking 2-methylnaphthalene biodegradation capabilities of fungal microbes with their genetic and potential metabolic attributes. The identified methylnaphthalene-degrading related genes include the P450 and dioxygenases such as *RHD* and *AROD*. Phylogenetic analysis and amino acid sequence comparisons of the identified dioxygenases suggest that these enzymes may serve previously unrecognized functions in 2-methylnaphthalene degradation. These findings underscore the genetic potential of largely uncharacterized fungal dioxygenases in the breakdown of organic pollutants. The widespread prevalence of these genes across fungal genomes suggests that the contribution of fungi to environmental pollutant removal is likely substantially underestimated. Consequently, further functional characterization of these dioxygenases is essential to uncover their specific roles and provide valuable insights into pollutant transformation mechanisms in complex environments.

A discovery of this study is that the fungal strain *Penicillium* sp. LJD-20 may employ multiple distinct mechanisms to remove pollutants. Given that the associated bacterium showed no direct involvement in pollutant metabolism, we did not further explore its degradation-related genes or pathways. We observed that fungi or fungal–bacterial consortia may employ dioxygenase systems and methyl-terminal oxidation pathways similar to those of bacteria to facilitate pollutant degradation. This finding substantially broadens our understanding of fungal metabolic pathways involved in the degradation of organic pollutants, particularly recalcitrant compounds. Previous studies have primarily focused on fungal assimilation of organic pollutants via cytochrome P450 enzymes [7, 65]. Additionally, some fungi are known to produce extracellular enzymes such as *Lac* and *MnP* to remove organic pollutants [23, 57]. Our research confirmed that LJD-20 possesses these conventional degradation capabilities. However, the occurrence of two 2-methylnaphthalene metabolic products suggested that strain LJD-20 may metabolize 2-methylnaphthalene *via* alternative metabolic routes. In particular, the identification of 2-naphthalenemethanol and 2-naphthaldehyde indicates the involvement of a methyl-terminal oxidation pathway (Fig. 6). Additionally, the presence of strain LJD-20 coincided with the appearance of other microorganisms potentially involved in methylnaphthalene removal. This phenomenon was only reported in some previous studies on bacterial bioaugmentation, highlighting the ecological interactions and synergistic effects in pollutant removal [66–68]. The LJD-20 genome harbors a diverse array of pollutant-degrading genes, including P450, *mhd*, *RHD*, and *AROD*, indicating its broad genetic capacity for versatile degradation. This metabolic potential may enable LJD-20 to efficiently process complex pollutants. Our study advances the genomic understanding of fungal biodegradation, particularly regarding emerging contaminants. By elucidating functional genes and potential microbial interactions, this study lays the foundation for harnessing fungal genetic diversity in bioremediation. These findings underscore the untapped potential of fungi in addressing environmental pollution and pave the way for innovative approaches to sustainable remediation.

## Conclusions

This study underscores the potential of fungi in the bioremediation of emerging organic pollutants. By integrating RACS–SIP with genomic analyses, we identified an active *Penicillium* sp. LJD-20 capable of *in situ* degradation of 2-methylnaphthalene. The genome of LJD-20 encodes a versatile array of degradation-related enzymes, including dioxygenases, methyl hydroxylases, and cytochrome P450s, underscoring its broad catabolic potential. Genomic and ecological evidence further suggest that *Penicillium* sp. LJD-20 may interact with surrounding microorganisms through potential metabolic complementarity and stress adaptation. Collectively, this work expands our understanding of fungal-mediated degradation of organic contaminants and provides a mechanistic and genomic foundation for harnessing fungal functional diversity in environmental bioremediation.

## Supplementary Material

Supporting_information_2025_10_wraf260

## Data Availability

All data needed to evaluate the conclusions in the paper are present in the paper and/or the Supplementary Materials.
