# Supplementary material for: In situ degradation of 2-methylnaphthalene by a soil Penicillium strain associated with fungal–bacterial interactions
Source: ISME J. 2025 Nov 25;19(1):wraf260. doi: 10.1093/ismejo/wraf260 (PMC12704424; doi:10.1093/ismejo/wraf260)
Supplement: Supporting_information_2025_10_wraf260 [file supporting_information_2025_10_wraf260.docx]

Supplementary Materials for

**​** ***In situ* Degradation of 2-Methylnaphthalene by a Soil *Penicillium* Strain Associated with Fungal-Bacterial Interactions**

Jibing Li^a,b,h^, Xixi Cai^c^, Menghui Li^a,b,h^, Dayi Zhang^d,e^, Bei Li^f^, Ling N. Jin^g^, Chunling Luo^a,b,h,^*, Gan Zhang^a,b^

^a^State Key Laboratory of Advanced Environmental Technology, Guangzhou Institute of Geochemistry, Chinese Academy of Sciences, Guangzhou 510640, China

^b^Guangdong Provincial Key Laboratory of Environmental Protection and Resources Utilization, Guangdong-Hong Kong-Macao Joint Laboratory for Environmental Pollution and Control, Guangzhou Institute of Geochemistry, Chinese Academy of Sciences, Guangzhou 510640, China

^c^Guangdong Key Laboratory of Ornamental Plant Germplasm Innovation and Utilization, Environmental Horticulture Research Institute, Guangdong Academy of Agricultural Sciences, Guangzhou 510640, China

^d^Key Laboratory of Groundwater Resources and Environment, Ministry of Education, Jilin University, Changchun 130012, China

^e^College of New Energy and Environment, Jilin University, Changchun 130021, China

^f^State Key Lab of Applied Optics, Changchun Institute of Optics, Fine Mechanics and Physics, Chinese Academy of Sciences, 130033, Changchun, China

^g^Department of Civil and Environmental Engineering; Department of Health Technology and Informatics, The Hong Kong Polytechnic University, Hung Hom, Kowloon 999077, Hong Kong

^h^University of Chinese Academy of Sciences, Beijing 100039, China

*Corresponding author: Dr. Chunling Luo

E-mail: [clluo@gig.ac.cn](mailto:clluo@gig.ac.cn) Tel.: +86-20-85290290; Fax: +86-20-85290706 Number of pages: 19

Number of tables: 5

Number of figures: 8

List of Abbreviations

| **Full Name** | **Abbreviation** |
| --- | --- |
| Raman-activated cell sorting | RACS |
| Stable isotope probing | SIP |
| Single-cell Raman spectroscopy | SCRS |
| Multiple displacement amplification | MDA |
| Relative enrichment factor | REF |
| Kyoto Encyclopedia of Genes and Genomes | KEGG |
| Minimal medium | MM |
| Bioaugmentation treatments with LJD-20 | BA treatments |
| Treatments without fungal supplement (Non-supplemented treatments) | NS treatments |
| Ring-hydroxylating dioxygenases | RHD |
| Aromatic ring-opening dioxygenases | *AROD* |
| Aldehyde dehydrogenase | *nidD* |

***Morphological Characterization, and Growth Optimization***

The morphological characteristics of LJD-20 were observed using transmission electron microscopy (TEM). Given the sensitivity of fungi to environmental factors such as temperature and pH, their effects on fungal growth and metabolic activity were quantitatively evaluated. Activated fungal cultures were transferred into 250-mL Erlenmeyer flasks containing 100 mL of PDB and incubated at 30°C with shaking at 150 rpm for 7 days. Harvested mycelia were washed, weighed, and adjusted to a concentration of ~10 g·L⁻¹ using sterile water. Mycelia were then homogenized using a tissue homogenizer to prepare fungal suspensions. These suspensions were inoculated onto PDA plates and incubated at six different temperature gradients: 18°C, 23°C, 28°C, 30°C, 33°C, and 38°C.For pH optimization, fungal suspensions were inoculated into PDB medium at varying pH levels (4–9) and incubated at 30°C. After 7 days of incubation, fungal growth was assessed by measuring mycelial biomass under each condition.

***DNA*** ***ultracentrifugation***

DNA extraction from each sample was performed using the PowerSoil DNA Isolation Kit (MO BIO, Carlsbad, CA) according to the manufacturer's instructions. Subsequently, the DNA obtained from the ^12^C_MP and ^13^C_MP microcosms was employed for CsCl gradient ultracentrifugation, following established protocols.[^1^](#_ENREF_1)^,^ [^2^](#_ENREF_2) Briefly, approximately 5 μg of DNA was mixed with a tris EDTA/CsCl solution at a buoyancy density (BD) of approximately 1.77 g/ml and loaded into Quick-Seal polyallomer tubes (13×51 mm, 5.1 mL, Beckman Coulter, Pasadena, CA, USA). Ultracentrifugation was carried out in a Beckman Coulter L-100XP ultracentrifuge at 47,500 rpm for 48 hours at 20 °C. Subsequently, 14 fractions were separated from each tube using a fraction recovery system (Beckman Coulter). The BD values of the separated DNA samples were determined using an AR200 digital refractometer, and DNA purification was achieved through glycogen-assisted ethanol precipitation.[^3^](#_ENREF_3)^,^ [^4^](#_ENREF_4) Based on buoyant density (BD) values, fractions with BD values of 1.7042–1.7089 g/mL were classified as “light” DNA, while those with BD values of 1.7342–1.7411 g/mL were classified as “heavy” DNA. Details are provided in the attachment.

***Chemical Analysis***

Chemical analyses were conducted on samples from different treatments at various time points to assess the degradation of methylnaphthalene in soil or MM medium. The analysis was performed using gas chromatography-mass spectrometry (GC-MS, Agilent 7890; Agilent Technologies, Santa Clara, CA, USA). To begin, 1000 ng of deuterated polycyclic aromatic hydrocarbons (PAHs) were introduced into the samples to serve as internal standards for assessing recovery efficiency. The samples were then extracted using a mixture of dichloromethane and acetone. The extracted samples were subjected to rotary evaporation for concentration and purified using silica/alumina columns. The final extract was concentrated to approximately 0.5 mL for further analysis. Prior to the instrumental analysis, 1000 ng of 2-fluorobiphenyl was added as an internal standard. methylnaphthalene analysis was conducted using negative chemical ionization (NCI) and selected ion monitoring (SIM). High-purity helium and methane were used as carrier and reaction gases, respectively.

To investigate the metabolic mechanisms of fungi on methylnaphthalene, we conducted a comprehensive analysis of methylnaphthalene metabolites in the MM medium treatment. After incubation, 100 mL of the sample was taken, and an equal volume of 70% methanol was added. The samples were vortexed for 15 minutes and then centrifuged at 12000 r/min for 3 minutes at 4°C. The supernatant was filtered through a 0.22 μm micropore filter and transferred to a vial for LC-MS/MS analysis. Chromatographic separation was achieved using a Waters ACQUITY Premier HSS T3 column (1.8 μm, 2.1 mm × 100 mm) with a mobile phase consisting of 0.1% formic acid in water (A) and 0.1% formic acid in acetonitrile (B). The column temperature was maintained at 40°C with a flow rate of 0.4 mL/min. Mass spectrometric detection was performed on an AB TripleTOF 6600 platform. The mass spectrometry parameters and collision energies are detailed in Table S3.

Additionally, we conducted enzyme activity assays to further explore the degradation mechanisms of functional microorganisms. Enzyme activities of laccase (*Lac*), lignin peroxidase (*LiP*), and manganese peroxidase (*MnP*) were measured for different treatments. Enzyme activity was determined according to the instructions provided with the corresponding assay kits (Beijing Solarbio Science & Technology Co., Ltd., Beijing, China). *Lac* activity was measured by absorbance at 420 nm, which corresponds to the degradation of the substrate 2,2’-azino-bis (3-ethylbenzothiazoline-6-sulfonate) diammonium salt (ABTS). *LiP* activity was determined by absorbance at 310 nm, which corresponds to the oxidation of veratryl alcohol. *MnP* activity was assessed by absorbance at 465 nm, which corresponds to the formation of tetramethoxyphenol. Prior to the assay, samples were centrifuged at 4500 rpm for 50 minutes at 4°C. The supernatant was mixed with the substrate, and the absorbance of the mixture was measured using a spectrophotometer.

***Statistical Analysis***

All experiments were conducted independently with three biological replicates. Statistical analyses were performed using analysis of variance (ANOVA) and independent sample t-tests. Data processing was carried out using R and Origin 8.0.25 software. Phylogenetic information of the active degrading bacteria was obtained through BLAST analysis (National Center for Biotechnology Information, USA). Human contamination (Blastn E-value threshold ≤ 10−5, bitscore ≥ 50, percent identity ≥ 75%) was further excluded by aligning reads to the human reference genome (build 37) using bowtie2 (version 2.1.0).[^5^](#_ENREF_5) Statistical significance was determined using SPSS software (version 24.0; IBM, USA), where p-values < 0.05 and < 0.001 were considered statistically significant and highly significant, respectively. Data are presented as mean ± standard deviation (SD). Mass spectrometry data of metabolites were converted to mzXML format using ProteoWizard, and peak extraction, alignment, and correction were performed with XCMS. Peaks with a missing rate greater than 50% were filtered out, and blank values were imputed before peak areas were normalized using support vector regression (SVR). Metabolite identification was performed by integrating laboratory databases, public databases, and the metDNA method, selecting substances with a comprehensive score > 0.5 and a QC sample coefficient of variation (CV) < 0.5.

**References**

1. Li, J. et al. Novel bacteria capable of degrading phenanthrene in activated sludge revealed by stable-isotope probing coupled with high-throughput sequencing. *Biodegradation* **28**, 423-436 (2017).

2. Li, J. et al. The catabolic pathways of in situ rhizosphere PAH degraders and the main factors driving PAH rhizoremediation in oil-contaminated soil. *Environ. Microbiol.* **23**, 7042-7055 (2021).

3. Li, J., Zhang, D., Li, B., Luo, C. & Zhang, G. Identifying the Active Phenanthrene Degraders and Characterizing Their Metabolic Activities at the Single-Cell Level by the Combination of Magnetic-Nanoparticle-Mediated Isolation, Stable-Isotope Probing, and Raman-Activated Cell Sorting (MMI–SIP–RACS). *Environ. Sci. Technol.* **56**, 2289-2299 (2022).

4. Bao, J. et al. New insight into the mechanism underlying the effect of biochar on phenanthrene degradation in contaminated soil revealed through DNA-SIP. *J. Hazard. Mater.* **438**, 129466 (2022).

5. Langmead, B., Trapnell, C., Pop, M. & Salzberg, S.L. Ultrafast and memory-efficient alignment of short DNA sequences to the human genome. *Genome Biol.* **10**, R25 (2009).


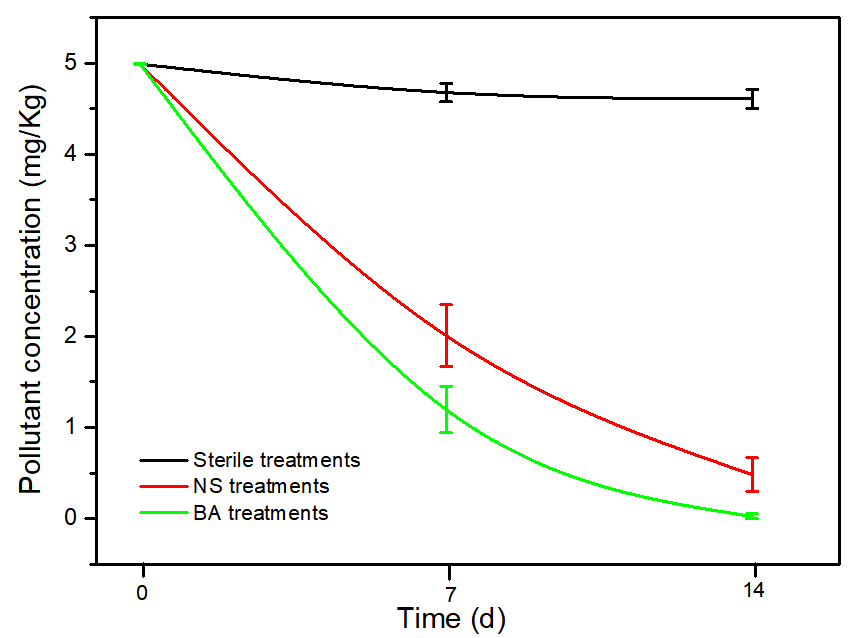


**Figure S1.** Biodegradation of 2-methylnaphthalene in each treatment after 7 and 14 days of incubation. Data are means of three replicates. NS refers to native soil treated with antibiotics but without the addition of *Penicillium* sp. LJD-20; BA represents soil inoculated with fungus LJD-20.

**
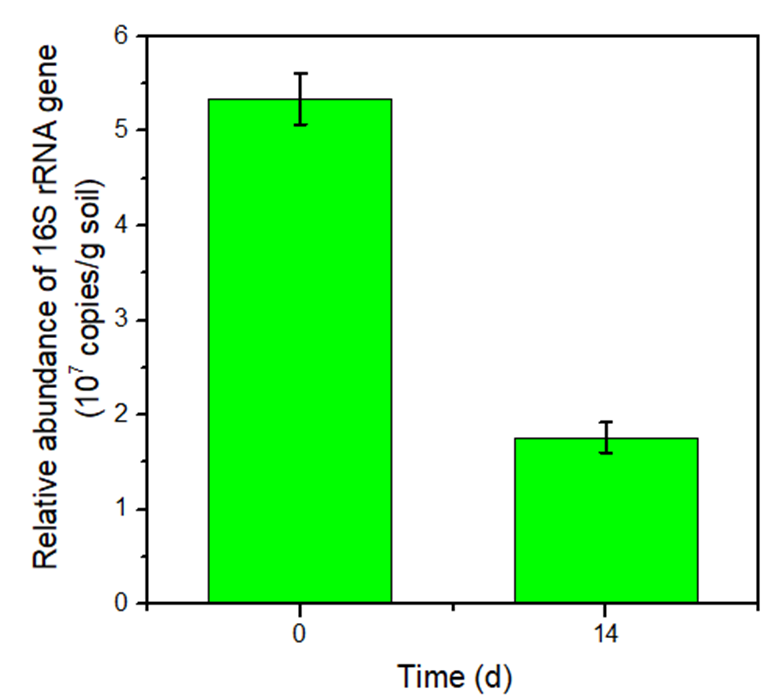
**

**Figure S2.** Relative abundance of 16S rRNA genes in samples from day 0 and day 14 of incubation under the NS treatment.


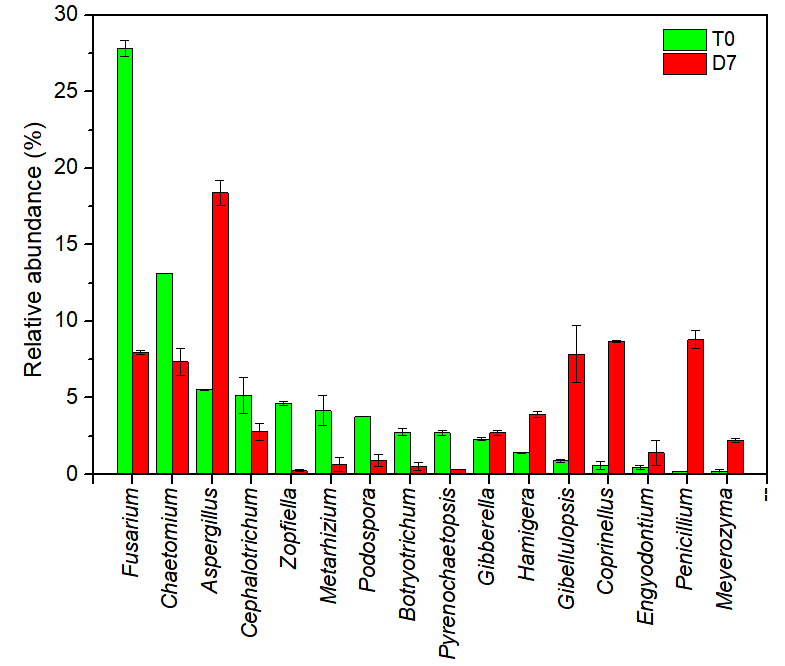


**Figure S3.** Relative abundance of fungal genera in different microcosms. Genera with a minimum relative abundance above 1% are shown. T0 represents the microbial community in the raw soil sample, while D7 represents the community in treated soil after 7 days of incubation.


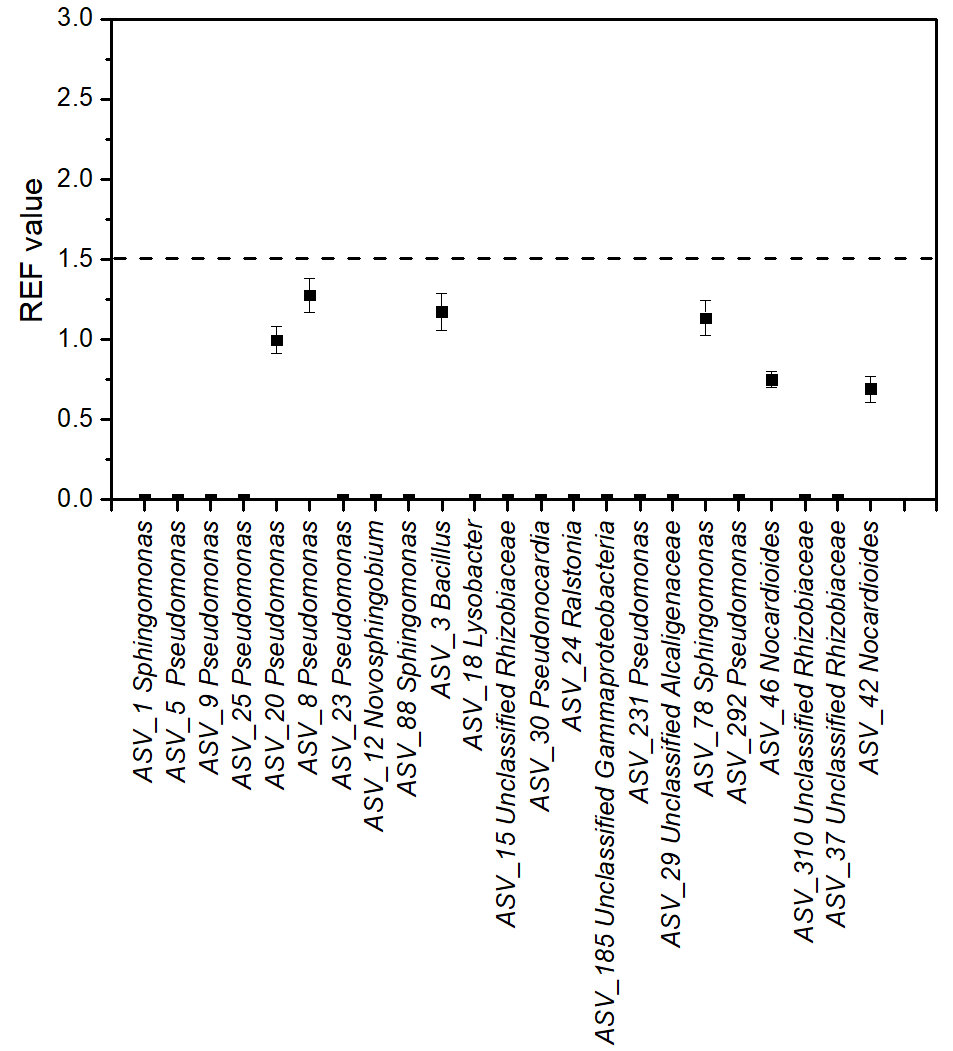


**Figure S4**. The enrichment factor (REF) of bacterial ASVs from the NS treatments.

**
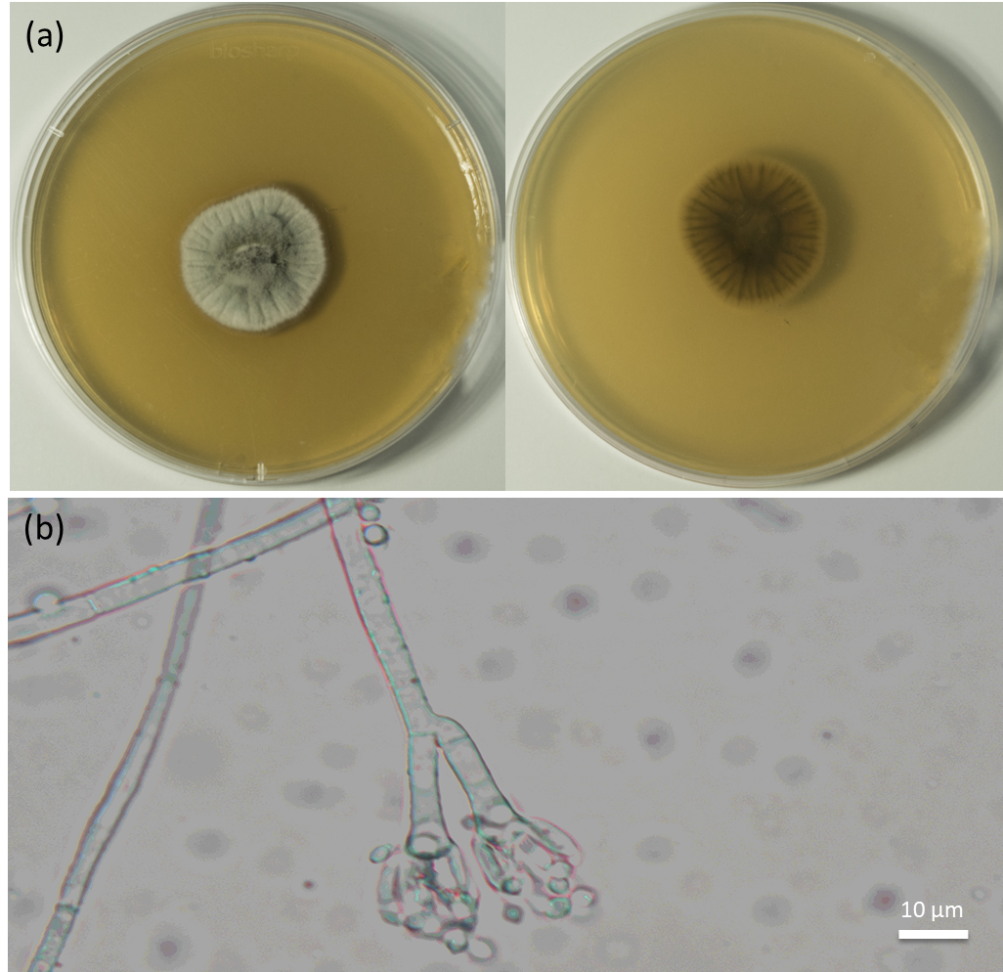
**

**Figure S5.** Morphology of strain LJD-20. (a) Photograph of strain LJD-20 on PDA plate. (b) Electron micrographs of cells of strain LJD-20; scale bar is 10 μm.


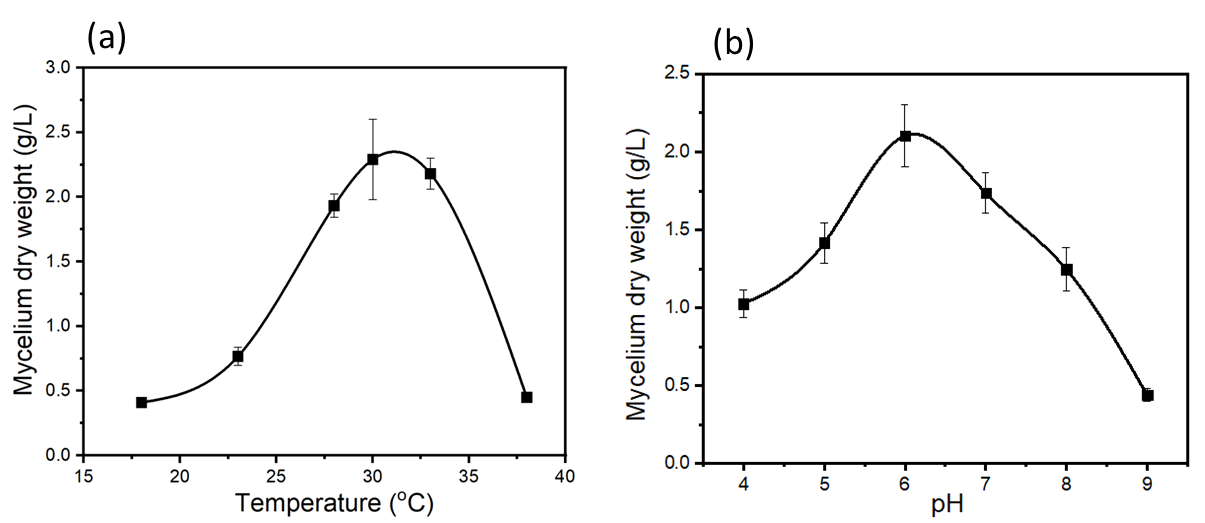


**Figure S6.** Growth curves of strain LJD-20 under various conditions after 7 days of cultivation in PDB medium: (a) at different temperatures, (b) at different pH levels.
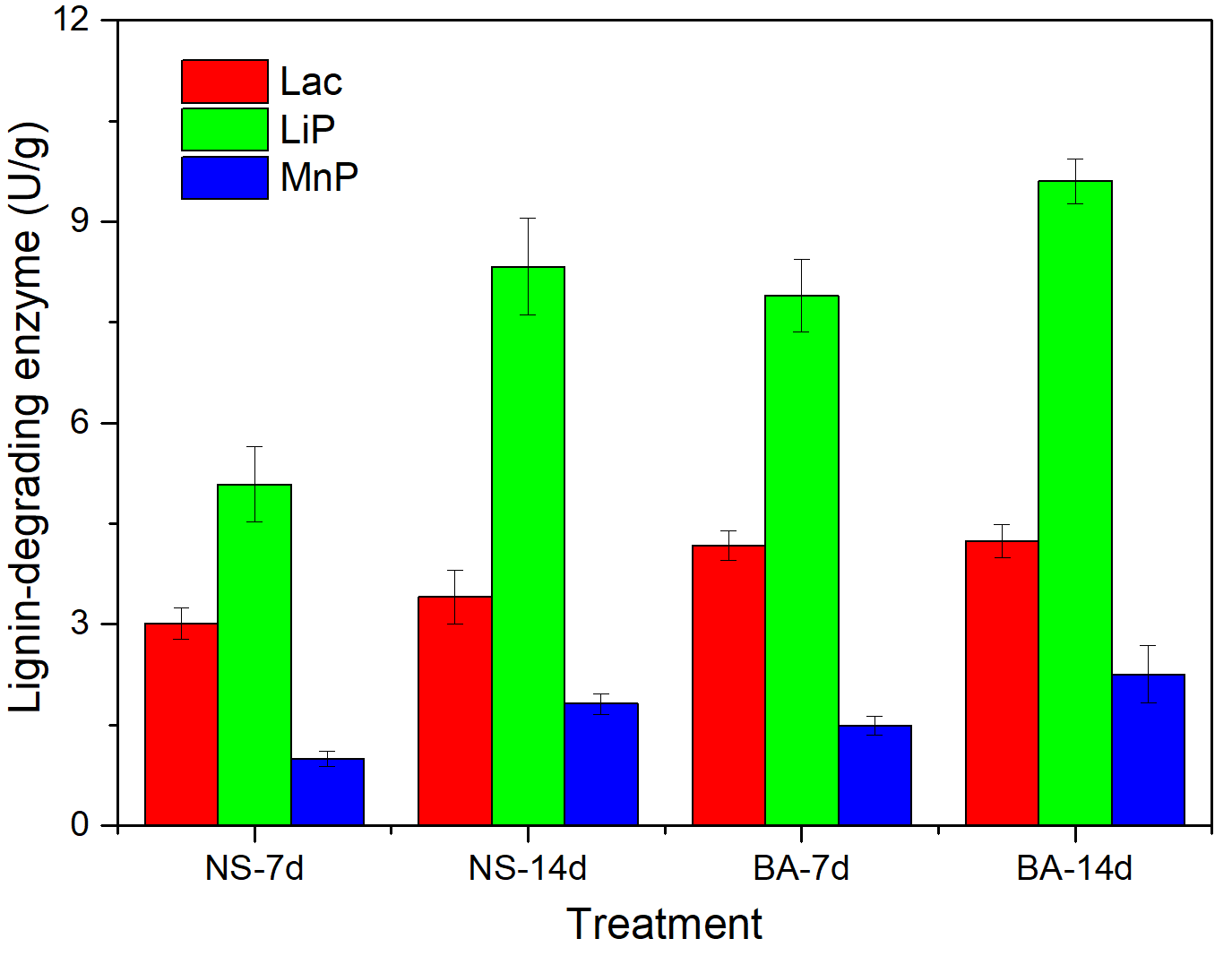


**Figure S7.** The laccase, manganese peroxidase, and lignin peroxidase activities of the soil in each treatment after 14 days of incubation, respectively. NS refers to native soil treated with antibiotics but without the addition of *Penicillium* sp. LJD-20; BA represents soil inoculated with fungus LJD-20.

**
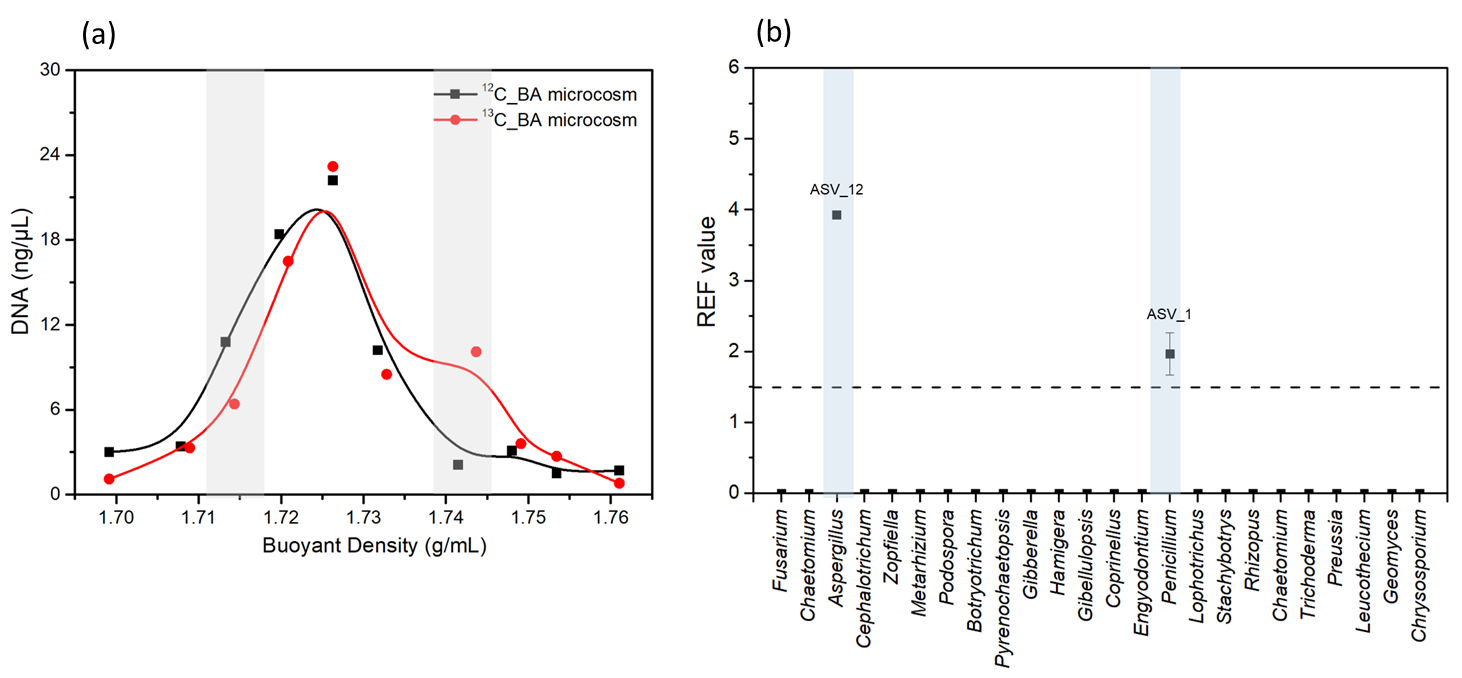
**

**Figure S8.** (a) Correlation between DNA concentration and buoyant density (BD, g/mL) in DNA extracted from the ^12^C_BA and ^13^C_BA microcosms. The “heavy” DNA fraction is highlighted. (b) The enrichment factor (REF) of fungal ASVs from the BA microcosms.

**Table S1.** Soil characteristics and 2-methylnaphthalene content in soil, data are presented as mean of three repetitions.

| **Soil characteristics** | **Content** |
| --- | --- |
| OM | 35.3 (mg/Kg) |
| TN | 0.96 (mg/Kg) |
| TP | 0.44 (mg/Kg) |
| TK | 14.3 (mg/Kg) |
| AN | 32.3 (mg/Kg) |
| AP | 2.58 (mg/Kg) |
| AK | 220.6 (mg/Kg) |
| Cu | 4.77 (mg/Kg) |
| Zn | 6.17 (mg/Kg) |
| Fe | 17.9 (mg/Kg) |
| Mn | 5.25 (mg/Kg) |
| Salt | 2.21 (mg/Kg) |
| 2-methylnaphthalene | 57.1 (ug/Kg) |

**Table S2.** Primers used for the PCR of ITS and 16S rRNA genes

| **Primer** | | **Sequence (5’-3’)** |
| --- | --- | --- |
| ITS | ITS3F | CCGCATCGATGAAGAACGCAGC |
|  | ITS4R | TCCTCCGCTTATTGATATGC |
| 16S rRNA | 515F | GTGCCAGCMGCCGCGGTAA |
|  | 806R | AACGCACGCTAGCCGGACTACVSGGGTATCTAAT |

**Table S3.** Mass spectrometry conditions for AB TripleTOF 6600.

| **Name** | **ESI+** | **ESI-** |
| --- | --- | --- |
| Duration (min) | 10 | 10 |
| IonSpray Voltage (V) | 5000 | -4000 |
| Temperature (°C) | 550 | 450 |
| lon Source Gas1 (psi) | 50 | 50 |
| lon Source Gas2 (psi) | 60 | 60 |
| Curtain Gas (psi) | 35 | 35 |
| Declustering Potential (V) | 60 | -60 |
| MS1 Collision Energy (V) | 10 | -10 |
| MS2 Collision Energy (V) | 30 | -30 |
| Collision Energy Spread (V) | 15 | 15 |

**Table S4**. The information of the assembled bins from bacteria within the symbiont.

| **Completeness (%)** | **Contamination (%)** | **GC** | **lineage** |
| --- | --- | --- | --- |
| 51.8 | 0 | 0.68 | d__*Bacteria*;p__*Proteobacteria*;c__*Gammaproteobacteria*;o__*Burkholderiales*;f__*Burkholderiaceae*;g__*Achromobacter* |

**Table S5.** Information on potential metabolic products.

| **Compounds** | **Formula** | **Mode** | **Molecular weight (Da)** | **RT (min)** | **Mass error** | **Level** | **score** | **LC mode** |
| --- | --- | --- | --- | --- | --- | --- | --- | --- |
| 2-Naphthalenemethanol | C11H10O | T3_positive | 158.0732 | 6.1725 | 2.891201 | 2 | 0.8439 | rp |
| 2-Naphthaldehyde | C11H8O | T3_positive | 156.0575 | 5.2191 | 2.065441 | 2 | 0.8035 | rp |
